# Precision nutrition targeting the gut microbiota for weight management: mechanisms and applications

**DOI:** 10.3389/fmicb.2026.1806152

**Published:** 2026-05-22

**Authors:** Liyuan Jin, Yuan Zhu, Yaozhe Ying, Pengcheng Yan, Huanzhi Jin, Zheyan Chen

**Affiliations:** 1 Department of General Practice, Wenzhou Third Clinical Institute Affiliated to Wenzhou Medical University, The Third Affiliated Hospital of Shanghai University, Wenzhou People's Hospital, Wenzhou, China; 2 Department of Clinical Nutrition Center, Wenzhou Third Clinical Institute Affiliated to Wenzhou Medical University, Wenzhou People's Hospital, Wenzhou, China; 3 School of Traditional Chinese Medicine, Wenzhou Medical University, Wenzhou, Zhejiang, China; 4 Department of Plastic and Reconstructive Surgery, Wenzhou Third Clinical Institute Affiliated to Wenzhou Medical University, The Third Affiliated Hospital of Shanghai University, Wenzhou People's Hospital, Wenzhou, China

**Keywords:** artificial intelligence, energy metabolism, genetic interactions, gut microbiota, multi-omics, obesity, precision nutrition, weight management

## Abstract

The gut microbiota, a highly individualized microbial community, is increasingly recognized for its role in weight regulation and metabolic health. This narrative review examines the bidirectional associations between gut microbiota and weight management, focusing on mechanisms involving energy metabolism, immune modulation, inflammatory responses, and appetite control. We also explore the interplay between gut microbiota and host genetic/epigenetic factors in obesity development. The review discusses how precision nutrition interventions may modulate gut microbiota composition to support personalized weight management strategies. Furthermore, we address the integration of artificial intelligence and multi-omics technologies, outlining current challenges and future directions. This review aims to provide a theoretical foundation for translating gut microbiota-targeted precision nutrition into clinical and research settings for effective weight management.

## 1 Introduction

Obesity has emerged as a significant global health crisis, characterized by an excessive accumulation of body fat that poses serious health risks, including cardiovascular disease, type 2 diabetes, and various metabolic disorders (Yang et al., 2022). This multifactorial chronic disease is influenced by a complex interplay of genetic, environmental, lifestyle, and microbial factors, which contribute to its pathogenesis and progression (Młynarska et al., 2025). The mechanisms underlying obesity are intricate and vary significantly among individuals, highlighting the need for a nuanced understanding of its etiology. Recent studies have emphasized the role of gut microbiota as one of the crucial environmental factors in the development of obesity, suggesting that the composition and functionality of the gut microbiome can significantly influence energy metabolism, appetite regulation, and inflammatory processes (Lorenzo et al., 2025).

The gut microbiome, a diverse community of microorganisms residing in the gastrointestinal tract, plays a pivotal role in maintaining metabolic homeostasis. Dysbiosis has been linked to obesity and its comorbidities through mechanisms including altered energy harvest, systemic inflammation, and impaired insulin sensitivity (Kamal et al., 2023). For instance, the relative abundance of bacterial phyla such as *Firmicutes* and *Bacteroidetes* differs between obese and non-obese individuals, with a higher Firmicutes-to-Bacteroidetes ratio frequently observed in obesity (Vishwakarma et al., 2025). Furthermore, gut-derived metabolites, particularly short-chain fatty acids (SCFAs), are implicated in body weight regulation and metabolic health, reinforcing the importance of gut microbiota in obesity management (Ramos-Lopez et al., 2024).

Precision nutrition—tailoring dietary interventions to individual genetic, metabolic, and gut microbial profiles—has emerged as a promising strategy for obesity management (Carvalho et al., 2025b). Diets rich in fiber or resembling the Mediterranean pattern have been shown to favorably modulate gut microbiota composition, promote beneficial bacteria, and enhance SCFA production, thereby aiding weight control and reducing inflammation (Ulusoy-Gezer and Rakicioglu, 2024). The integration of omics technologies (genomics, metabolomics, and microbiomics) enables comprehensive profiling of individual responses to dietary interventions, facilitating the identification of microbial and genetic signatures that inform personalized recommendations (Alsanie, 2026). Despite these advances, translating such insights into clinical practice faces persistent challenges, including inter-individual variability in dietary responses and the need for large-scale, long-term validation studies (Balbino et al., 2026).

The intricate relationship between gut microbiota and obesity underscores the necessity of a multifaceted, personalized approach to weight management that accounts for individual biological and environmental factors. Continued research into precision nutrition, coupled with a deeper mechanistic understanding of the gut microbiome, holds promise for developing effective strategies to combat obesity and improve metabolic health. Future efforts should prioritize elucidating the causal pathways linking gut microbiota to host metabolism to guide evidence-based interventions. The evidence cited in this article includes large-scale randomized controlled trials (RCTs), observational cohort studies, and mechanistic animal experiments. Among these, RCTs provide the highest level of evidence, whereas findings from animal experiments should be extrapolated to humans with caution.

## 2 Physiological mechanisms linking gut microbiota to weight regulation

### 2.1 The impact of gut microbiota on host energy metabolism

The gut microbiota influences host energy metabolism primarily through fermentation of dietary fibers into short-chain fatty acids (SCFAs), including acetate, propionate, and butyrate. These SCFAs serve as energy substrates for intestinal epithelial cells and modulate metabolic pathways. For example, butyrate has been associated with enhanced fatty acid oxidation and lipid metabolism, potentially affecting the host's energy balance (Liu et al., 2024a). SCFAs are also involved in signaling pathways that influence appetite regulation and energy expenditure (Mohammadi et al., 2020).

Microbial communities can affect the efficiency of energy extraction from food. For example, studies have demonstrated that individuals with a higher abundance of specific bacterial taxa, such as *Bacteroidetes*, tend to have a more efficient energy extraction capability compared to those with a predominance of *Firmicutes* (Lee and Lee, 2020). This differential energy extraction can significantly impact weight management and obesity development, as those with a microbiota composition favoring energy-dense extraction may be more prone to weight gain and metabolic disorders (Igudesman et al., 2025).

The relationship between gut microbiota composition and energy metabolism is further complicated by the observation that obesity is often associated with reduced microbial diversity. Studies in high-fat-diet-induced obese aged mice indicate that obesity frequently leads to lower gut microbiota diversity, which correlates with altered metabolic profiles and impaired energy metabolism (Li et al., 2025a). Lower microbial diversityis associated with the promotion of obesity through loss of beneficial species (e.g., *Faecalibacterium, Roseburia* from *Firmicutes*, and *Bacteroidetes*). Their depletion reduces SCFAs, increases gut permeability and low-grade inflammation, and boosts energy extraction—driving fat accumulation.

Diet accelerates this: high-fat diets raise *Firmicutes*/*Bacteroidetes* ratio (phylum), lower *Clostridia* (class, especially *Lachnospiraceae*), and raise *Erysipelotrichia*. Ultra-processed foods cut beneficial families (*Lachnospiraceae, Ruminococcaceae*) and raise pro-inflammatory ones (*Enterobacteriaceae, Proteobacteria*). These changes deepen diversity loss, creating an energy imbalance cycle that worsens obesity (Borrego-Ruiz and Borrego, 2025).

### 2.2 Gut microbiota, immunity, and inflammatory response

**Figure 1:**
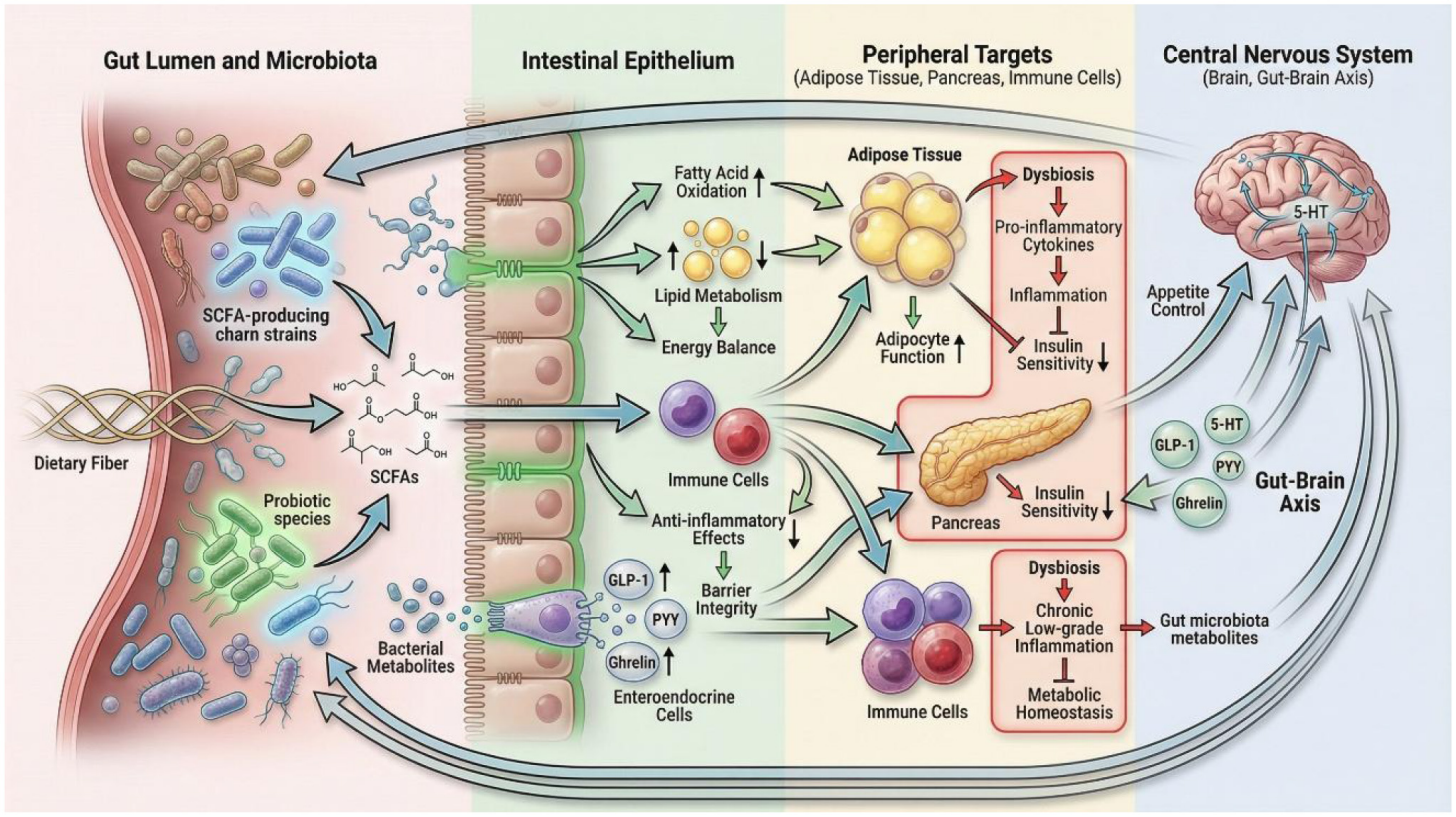
Schematic representation of the gut-brain axis and its role in weight regulation. This figure illustrates the interactions between gut microbiota, dietary components, host metabolism, and the central nervous system, highlighting pathways involving short-chain fatty acids (SCFAs), appetite hormones, and immune signaling.

The gut microbiota is closely associated with the maintenance of immune homeostasis and the modulation of inflammatory responses. Dysbiosis of the gut microbiota has been linked to chronic low-grade inflammation, which in turn correlates with impaired adipose tissue function and reduced insulin sensitivity, thereby contributing to obesity-related metabolic disorders (Ezenabor et al., 2024). Dysbiosis, characterized by an imbalance in microbial composition, is associated with increased intestinal permeability, which may facilitate the translocation of lipopolysaccharides (LPS) and other pro-inflammatory agents into the bloodstream, potentially triggering systemic inflammation (Park et al., 2024). This inflammatory state has been implicated in the development of insulin resistance and metabolic syndrome. Emerging evidence from observational studies suggests that alterations in gut microbiota composition are associated with enhanced inflammatory responses and metabolic dysregulation (Gopal et al., 2024). Furthermore, chronic low-grade inflammation is recognized as a significant factor in the pathogenesis of obesity, as it may promote a vicious cycle of metabolic dysfunction and further dysbiosis, ultimately exacerbating the condition (Sasidharan Pillai et al., 2024). While these associations are well-documented, it is important to note that the causal directionality between gut microbiota alterations and metabolic disorders has not been firmly established in controlled human studies (Piwchan et al., 2025) (Figure 1).

Probiotics and SCFA-producing bacterial strains have been shown to modulate immune responses and reduce inflammatory markers in some studies. SCFAs can enhance gut barrier function, regulate immune cell activity, and lower inflammatory markers (Wang et al., 2023b). For example, SCFAs influence the differentiation and function of regulatory T cells, which help maintain immune tolerance (Scarpellini et al., 2024). Administration of certain probiotics has been associated with improved metabolic profiles and reduced inflammatory markers in obese individuals, suggesting that targeted microbiome modulation may hold therapeutic potentia (Ding et al., 2025).

The activation of immune-inflammatory pathways is closely linked to the integrity of the gut barrier, and the restoration of microbiota balance can significantly aid in the recovery of this barrier function. Disruption of the gut barrier, often referred to as “leaky gut,” allows for the passage of microbial products and toxins into the systemic circulation, which can activate immune responses and lead to chronic inflammation (Wang and Gong, 2022). Interventions aimed at restoring gut microbiota, such as dietary modifications, prebiotics, and probiotics, have shown promise in enhancing gut barrier integrity and reducing inflammation (Chénard et al., 2020). For instance, studies have demonstrated that diets rich in fiber can promote the growth of beneficial bacteria that produce SCFAs, thereby reinforcing the gut barrier and mitigating inflammatory responses (Wastyk et al., 2021). Future research should continue to explore the mechanisms through which gut microbiota influence immune and inflammatory pathways, with the aim of developing targeted interventions for obesity and related metabolic diseases. Understanding these complex interactions may pave the way for innovative therapeutic strategies that harness the gut microbiome to promote health and prevent disease (Wang et al., 2023c).

### 2.3 The role of gut microbiota in appetite and neuroendocrine regulation

Gut microbiota influences appetite-regulating hormones such as glucagon-like peptide-1 (GLP-1), peptide YY (PYY), and ghrelin. These hormones are secreted by enteroendocrine cells in response to nutrients, and their secretion is modulated by microbial metabolites including SCFAs, which can stimulate GLP-1 and PYY release, promoting satiety and reducing food intake (Han et al., 2021). Conversely, alterations in gut microbiota composition observed in obesity may be associated with dysregulation of these hormones, potentially leading to increased appetite (Yu et al., 2024).

The gut-brain axis provides a pathway for microbial influence on central appetite regulation. Microbial metabolites can cross the blood-brain barrier or signal via the vagus nerve, impacting appetite and energy expenditure (Ribeiro et al., 2025). For example, SCFAs can cross the blood-brain barrier via monocarboxylate transporters, or can activate vagal afferent neurons through GPR41 and GPR43 receptors, thereby modulating hypothalamic appetite-regulating circuits and influencing the release of neurotransmitters such as serotonin and GABA (Qi et al., 2024). Additionally, gut microbiota influences synthesis of neurotransmitters such as serotonin and gamma-aminobutyric acid (GABA), which are involved in mood and appetite regulation (Li et al., 2024). Certain microbial populations have been associated with changes in hypothalamic neuropeptide expression, which is critical for appetite control (Romaní-Pérez et al., 2025). Studies have shown that specific microbial populations, such as *Limosilactobacillus reuteri* and *Bifidobacterium longum*, can influence the expression of neuropeptides in the hypothalamus, which are critical for appetite control (Zhang et al., 2024a). This indicates that the gut microbiota may serve as a modulator of not only hormonal signals but also neuroendocrine pathways that govern feeding behavior.

## 3 The role of genetic and epigenetic factors in interaction with gut microbiota in weight management

### 3.1 Interaction between obesity-related genetic variants and gut microbiota

**Figure 2:**
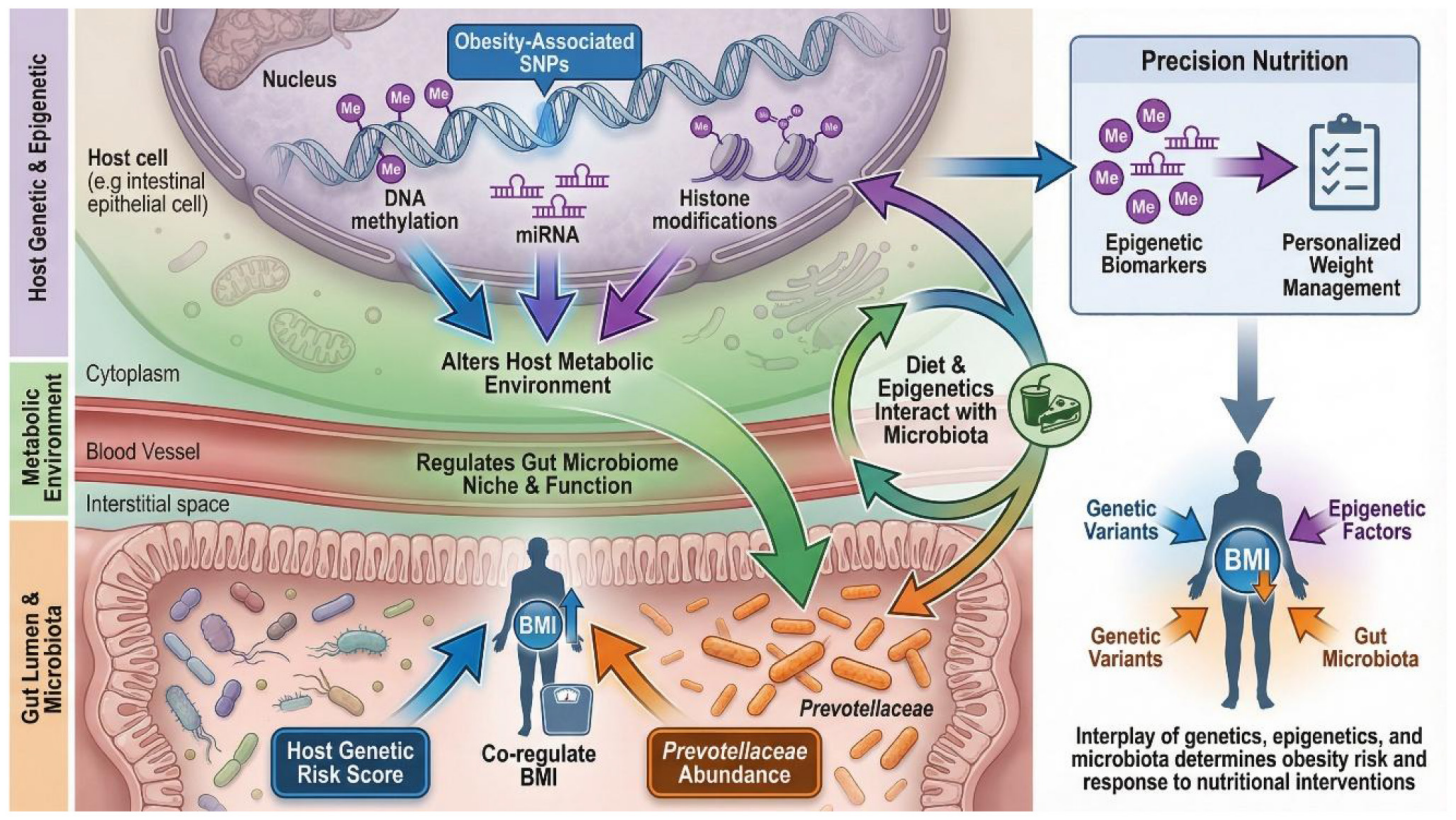
Interplay between host genetics, epigenetics, and gut microbiota in obesity.

The interplay between genetic predispositions and gut microbiota composition has garnered significant attention in recent obesity research. Numerous studies have identified single nucleotide polymorphisms (SNPs) associated with obesity, revealing that certain genetic variants can influence the abundance and diversity of gut microbiota. For instance, a genetic risk score derived from obesity-related SNPs has been shown to correlate with specific microbial families, such as Prevotellaceae, which is particularly notable in women. This association suggests that genetic background may shape the gut microbiome, thereby affecting an individual's susceptibility to weight gain and obesity-related metabolic disorders (Guo et al., 2022). Furthermore, in women, higher Prevotellaceae abundance interacts with an elevated genetic risk score to increase BMI, illustrating a convergence of microbial and genetic factors in body weight regulation and supporting personalized obesity management strategies targeting both the gut microbiome and host genetics (Mansour et al., 2024). The mechanism underlying this interaction appears to be multifaceted. Genetic factors may alter the metabolic environment of the host, which in turn can shape the ecological niche and functional capabilities of the gut microbiota. For example, specific genetic variants can affect the host's metabolism, leading to changes in the availability of substrates for gut bacteria, which may promote the growth of certain microbial populations over others. This dynamic interplay can result in a feedback loop where the gut microbiota further influences metabolic pathways, thereby exacerbating or alleviating obesity risk (Cheng et al., 2023). Moreover, the presence of certain SNPs may predispose individuals to dysbiosis, characterized by an imbalance in microbial communities that can lead to increased inflammation and metabolic dysfunction, further complicating weight management efforts (Figure 2).

Additionally, the role of gut microbiota in mediating the effects of genetic predispositions on obesity is supported by findings from studies utilizing animal models. For instance, research has demonstrated that specific genetic backgrounds can lead to distinct microbiota profiles that correlate with variations in body weight and metabolic health. These findings underscore the importance of considering both genetic and microbial factors in understanding obesity's etiology and developing therapeutic interventions (Sehgal et al., 2022). The emerging field of nutrigenomics, which examines how genetic variations affect responses to dietary interventions, is particularly relevant here. By integrating insights from genomics and microbiome research, it may be possible to create more effective, tailored dietary recommendations that account for an individual's genetic predisposition and microbiota composition.

### 3.2 Epigenetic regulation and interaction with gut microbiota

The interplay between diet-induced epigenetic modifications and gut microbiota is a burgeoning area of research that highlights the complex mechanisms influencing fat metabolism and energy balance. Dietary components can lead to significant changes in DNA methylation, histone modifications, and microRNA (miRNA) expression, which in turn can affect the composition and function of the gut microbiota. For instance, fermentable fibers (e.g., inulin) promote *Bifidobacterium* growth through enhanced short-chain fatty acid (SCFA) production, while polyphenol-rich foods stimulate *Akkermansia muciniphila* via improved mucin turnover (Van den Abbeele et al., 2023). In contrast, high-fat Western diets favor *Bilophila wadsworthia* expansion by increasing bile-tolerant substrate availability (Rodríguez-Daza and de Vos, 2022), collectively impacting metabolic homeostasis (Sayavedra et al., 2025). These SCFAs can inhibit histone deacetylases, leading to increased histone acetylation and subsequent activation of genes involved in lipid metabolism and inflammation regulation (Woo and Alenghat, 2022). This intricate relationship underscores the importance of considering both dietary habits and gut microbiota when addressing obesity and metabolic disorders.

Epigenetic mechanisms play a pivotal role in the host's ability to adapt to environmental changes and nutritional interventions, thereby influencing weight regulation. Research has demonstrated that epigenetic modifications can affect gene expression patterns that govern metabolic processes, including those related to adipogenesis and energy expenditure. For example, alterations in DNA methylation patterns have been linked to obesity and metabolic syndrome, suggesting that the epigenome acts as a dynamic interface between environmental factors, such as diet and gut microbiota, and the host's metabolic phenotype (Tian and Chen, 2024). Furthermore, the gut microbiota itself can influence the host's epigenetic landscape, creating a feedback loop where changes in microbial composition can lead to further epigenetic modifications that perpetuate metabolic dysregulation. This bidirectional interaction emphasizes the need for a comprehensive understanding of how epigenetic factors mediate the effects of gut microbiota on host metabolism.

Looking ahead, the integration of epigenetic markers into precision nutrition strategies holds promise for optimizing individual weight management plans. By identifying specific epigenetic changes associated with dietary patterns and gut microbiota composition, personalized interventions can be developed that target these modifications to promote healthier metabolic outcomes. For instance, interventions that enhance the abundance of beneficial microbial species through dietary adjustments may not only improve gut health but also lead to favorable epigenetic changes that support weight loss and metabolic health (Narasimhan et al., 2025). The potential for utilizing epigenetic profiles as biomarkers for tailoring dietary recommendations could revolutionize approaches to obesity management, making them more effective and individualized. As research continues to unravel the complexities of the gut microbiota-epigenetics relationship, it is essential to explore how these insights can be translated into practical applications for improving health outcomes and preventing metabolic diseases.

## 4 Precision nutrition interventions targeting gut microbiota

### 4.1 Personalized dietary strategies based on gut microbiota for improving metabolic health

Precision nutrition, a burgeoning field, focuses on tailoring dietary interventions based on individual characteristics, including genetic, metabolic, and microbiome profiles. This personalized approach is particularly promising in managing chronic diseases such as diabetes, hypertension, and dyslipidemia. Recent studies have shown that dietary interventions based on gut microbiota characteristics can significantly improve metabolic parameters, including blood glucose, lipid levels, and blood pressure. For instance, specific dietary patterns such as the Mediterranean diet and plant-based diets, which promote the growth of beneficial gut bacteria, have been linked to enhanced insulin sensitivity and improved glycemic control in diabetic patients (Carvalho et al., 2025a). Moreover, the integration of precision nutrition strategies has been shown to yield better outcomes in managing chronic conditions compared to traditional one-size-fits-all dietary guidelines. By focusing on individual responses to food and nutrients, healthcare providers can develop more effective dietary recommendations that not only address the immediate health concerns of patients but also promote long-term metabolic health.

### 4.2 Characteristics of gut microbiota as a basis for personalized nutritional interventions

The gut microbiota plays a pivotal role in individual responses to dietary interventions. Specific microbial populations serve as predictive markers for personalized nutrition. For instance, individuals with a *Prevotella-dominant* enterotype exhibit better metabolic outcomes (e.g., greater weight loss and short-chain fatty acid production) on high-fiber diets compared to *Bacteroides-dominant* individuals, who may require alternative strategies (Hjorth et al., 2019). Mechanistically, Prevotella efficiently ferments fiber into propionate and butyrate, whereas *Bacteroides* prioritizes protein and fat metabolism. Additionally, higher abundance of butyrate-producing bacteria, such as *Faecalibacterium prausnitzii*, is associated with improved insulin sensitivity following fiber supplementation (Christensen et al., 2019). These findings underscore the importance of gut microbiota profiling to tailor effective dietary interventions for weight management and metabolic health.

Through analysis of microbial lineage and functional capabilities, researchers have attempted to develop targeted dietary strategies. For example, high-fiber dietary interventions have been associated with enhanced SCFA production and improved metabolic parameters, including better insulin sensitivity and reduced inflammation (Guan and Liu, 2023). However, most studies to date are small and lack prospective validation of microbiota-based dietary predictions.

### 4.3 The impact of dietary patterns on gut microbiota and body weight

Dietary patterns significantly influence the composition and functionality of the gut microbiota, which in turn plays a crucial role in weight management. The Mediterranean diet, characterized by high consumption of dietary fiber, healthy fats, and fermented foods, has been associated with the promotion of beneficial gut bacteria such as *Bifidobacterium* and *Lactobacillus*. These probiotics are known to enhance gut health and metabolic processes, thereby aiding in weight control and reducing the risk of obesity-related diseases (Ecarnot and Maggi, 2024). The high fiber content in this dietary pattern not only supports the growth of beneficial microbes but also facilitates the production of SCFAs, which are essential for maintaining gut integrity and regulating appetite (Zhang et al., 2025). In contrast, the consumption of ultra-processed foods and low-fiber diets has been linked to a decrease in microbial diversity, which may exacerbate obesity and metabolic disorders. Such diets often lack essential nutrients and fiber, leading to dysbiosis—a condition characterized by an imbalance in gut microbiota that can promote inflammation and insulin resistance (Chen et al., 2024). Studies have shown that individuals consuming high amounts of processed foods exhibit a higher prevalence of obesity and metabolic syndrome, highlighting the detrimental effects of poor dietary choices on gut health and body weight (Fu et al., 2024).

Moreover, nutritional supplements such as prebiotics and probiotics are emerging as adjuncts in weight management strategies. For weight control, preferred prebiotics include inulin, fructooligosaccharides (FOS), and short-chain fructo-oligosaccharides (scFOS), which have been shown to reduce body weight, BMI, and fat mass (Li et al., 2025b). Preferred probiotics include *Lactobacillus plantarum* and *Bifidobacterium* species; evidence indicates that these strains contribute to improvements in body weight and metabolic parameters (Huang and Cheng, 2025; Aleali et al., 2025). Clinical trials (30 obese children) have shown that combining dietary interventions with probiotic supplementation can lead to significant reductions in body weight and fat mass, particularly in overweight individuals (Zhou et al., 2024). However, the effectiveness of these interventions may vary based on individual microbiota profiles, suggesting that personalized nutrition approaches could optimize outcomes in weight management (Yamamoto-Wada et al., 2025).

### 4.4 Body fat distribution and gut microbiota

Body fat distribution—particularly visceral (central) adiposity—is a stronger predictor of metabolic risk than overall adiposity. Central obesity is associated with higher insulin resistance compared to peripheral fat distribution (Lee et al., 2022). Visceral fat secretes pro-inflammatory cytokines and adipokines that can promote systemic inflammation and insulin resistance (Steiner and Berry, 2022), whereas subcutaneous gluteofemoral fat may have a more favorable metabolic profile (Choppin et al., 2022).

Traditional metrics such as Body Mass Index (BMI) may not adequately reflect body fat distribution and its implications for health outcomes. Therefore, a more nuanced understanding of body fat distribution—including trunk fat mass, limb fat mass, and overall body fat percentage—is essential for developing effective dietary interventions. Precision nutrition strategies targeting high-risk fat deposits (e.g., trunk fat) could incorporate biomarker monitoring (e.g., C-reactive protein, triglyceride-glucose index) to assess individual responses (Bai et al., 2026). Dietary modifications that enhance gut microbiota diversity may improve lipid metabolism. For example, in a high-fat diet-induced obese mouse model, supplementation with *Lactobacillus paracasei* reduced body weight and fat accumulation while increasing beneficial bacteria (Liu et al., 2022). These findings from animal models require confirmation in human randomized controlled trials before clinical recommendations can be made.

## 5 Artificial intelligence and multi-omics in precision nutrition

### 5.1 AI-driven prediction of individual nutritional responses

The future of weight management and chronic disease prevention lies in integrating multi-omics and artificial intelligence (AI). Multi-omics (genomics, proteomics, metabolomics) reveals biological factors influencing metabolism; for example, it can identify gut microbiota profiles linked to obesity, enabling targeted interventions (Ni et al., 2023). AI analyzes large datasets to uncover patterns beyond traditional methods. Machine learning algorithms, such as Random Forest and Gradient Boosting, predict metabolic responses using microbiome, genetic, and dietary data (Belkhouribchia and Pen, 2025). Some models forecast postprandial glycemic responses, allowing tailored advice to reduce blood sugar spikes (Jeong et al., 2026). AI can also predict weight loss outcomes based on individual microbiota profiles (Liu et al., 2024b). Personalized dietary designs that incorporate microbiota modulation can be instrumental in targeting high-risk fat areas. For instance, a chronotype-adapted dietary pattern, which aligns meal timing with an individual's circadian rhythm, can modulate gut microbiota composition by promoting beneficial bacteria and SCFAs production, thereby influencing fat storage and metabolism and mitigating the adverse effects of excess trunk fat (Dinu et al., 2024).

In addition, AI and multi-omics streamline biomarker discovery for dietary responses (Su et al., 2025) and enable real-time monitoring with dynamic adjustments to nutrition plans. AI-driven analytics can identify environmental triggers (e.g., socioeconomic or cultural influences on diet) (Zhang et al., 2024b), creating a responsive care model that evolves with patient needs (Kim et al., 2025). Beyond individual care, AI also supports public health nutrition by detecting population trends and at-risk groups (Keohavong, 2025). Nevertheless, several limitations exist. Many lack prospective validation. The “black box” nature reduces interpretability and clinical trust. Real-time integration with wearables or apps (e.g., MealMeter) remains experimental (Arefeen et al., 2025). Data privacy, algorithmic bias, and equitable access remain unresolved (Ferreira et al., 2025). Thus, combining multi-omics and AI enables personalized, data-driven nutritional strategies. This approach improves individual outcomes and aids public health efforts against obesity. Ensuring accessibility across diverse populations is essential.

### 5.2 Multi-omics integration: potential and pitfalls

Integration of multi-omics technologies—metagenomics, metabolomics, and transcriptomics—offers a systems-level view of gut microbiota–host interactions. Metagenomics provides taxonomic and functional profiling of microbial communities (Cong and Endo, 2024); metabolomics identifies microbial and host metabolites (Sun et al., 2025); transcriptomics assesses gene expression changes (Kortesniemi et al., 2023). However, a major challenge in integrating these multi-omics data for precision nutrition is the substantial heterogeneity across gut microbiota studies. This heterogeneity arises from four sources: (1) differences in sequencing technologies-−16S rRNA amplicon sequencing provides genus-level resolution with limited functional inference, whereas whole-genome shotgun metagenomics offers species-level taxonomic and functional profiling at a higher cost; (2) variability in sample collection, storage, DNA extraction, and bioinformatics pipelines, which can introduce batch effects and reduce cross-study comparability; (3) diversity in dietary assessment methods (e.g., food frequency questionnaires, 24-h recalls, food diaries), each with distinct recall biases and measurement errors; and (4) population diversity in genetics, ethnicity, geography, and lifestyle, which limits the generalizability of microbial signatures identified in one cohort to another. Consequently, many reported associations between microbial features and metabolic outcomes remain dataset-specific and fail to replicate in independent populations. Addressing these sources of heterogeneity through standardized protocols, multi-cohort validation, and harmonized bioinformatics frameworks is essential for advancing precision nutrition from discovery to clinical application.

Machine learning applied to multi-omics data can uncover associations between microbiome profiles and nutritional responses (Shalish et al., 2025). Some studies have identified microbial signatures associated with favorable metabolic responses to specific foods or dietary patterns. However, these associations are often dataset-specific and fail to replicate across different populations. The clinical translation of multi-omics integration into precision nutrition remains in early stages (Russo et al., 2023; Vancamp et al., 2024; Khusainova et al., 2025).

### 5.3 Data privacy, ethical considerations, and challenges

The use of big data and AI in personalized nutrition raises significant ethical and practical concerns. These include: (1) Data privacy: Gut microbiome, genetic, and metabolic data are highly sensitive. Robust data protection measures are essential (Mundt et al., 2025). (2) Algorithmic bias: Models trained on non-representative populations may produce inaccurate or harmful recommendations for underrepresented groups. (3) Lack of standardization: No consensus exists on which microbial features, sequencing methods (16S rRNA vs. shotgun), or bioinformatics pipelines should be used for clinical decision-making. (4) Regulatory gaps: No approved AI-based nutrition recommendation systems currently exist for clinical use. Rigorous clinical validation and ethical oversight are needed before implementation (Gao et al., 2023).

## 6 Clinical application of gut microbiota targeted precision nutrition in obesity prevention and treatment

### 6.1 Gut microbiota characteristics in obesity and intervention responses

Obese individuals often exhibit reduced gut microbial diversity compared to lean controls (Liu et al., 2021). Inter-individual variability in gut microbiota composition influences responses to weight loss interventions, with some studies reporting that individuals with distinct baseline microbiota profiles respond differently to the same dietary changes (Angelini et al., 2024; Delzenne and Rodriguez, 2022). This observation supports the rationale for personalized nutrition; however, prospective studies that use baseline microbiota to successfully predict weight loss outcomes are still scarce.

Dietary interventions can induce significant changes in gut microbiota composition, which correlate with weight loss and improved metabolic markers. For instance, a randomized controlled trial found that participants who underwent a dietary intervention experienced improvements in gut microbiota diversity and composition, associated with reductions in body weight and waist circumference (Zheng et al., 2022). Interventions such as probiotics, prebiotics, and dietary modifications have also been shown to favorably modulate gut microbiota and enhance metabolic outcomes (Sheykhsaran et al., 2023).

Physical activity influences gut microbiota diversity as well. Some randomized controlled trials have demonstrated that exercise interventions can increase beneficial microbial species and contribute to weight loss (Wang et al., 2023a). The synergistic effects of diet and exercise on gut microbiota highlight the potential of comprehensive lifestyle interventions to enhance weight management strategies. Future research should continue to investigate specific microbial targets and mechanisms involved in the interplay between gut microbiota, diet, and metabolic health. Such knowledge will be crucial for developing tailored interventions aimed at combating obesity and its associated comorbidities.

### 6.2 The role of gut microbiota in obesity-related chronic diseases

The gut microbiota plays a critical role in the pathogenesis of obesity-related chronic diseases such as diabetes, hypertension, and cardiovascular diseases. Dysbiosis, or an imbalance in gut microbiota composition, has been closely linked to the development of these metabolic disorders. For instance, obesity is often characterized by an increased Firmicutes/Bacteroidetes ratio, which has been associated with greater energy harvest from the diet and subsequent weight gain (Li et al., 2023). This imbalance can lead to an overproduction of endotoxins, such as lipopolysaccharides (LPS), which can trigger systemic inflammation and contribute to insulin resistance, a precursor to type 2 diabetes (Lin et al., 2022). In addition, the gut microbiota can influence host metabolic processes through the production of short-chain fatty acids (SCFAs) and other metabolites involved in energy metabolism and inflammation regulation (Lu et al., 2025).

Modulation of gut microbiota diversity has been shown to improve metabolic parameters and reduce disease risk. For example, interventions that enhance microbial diversity, such as dietary changes or the use of probiotics, have been associated with improved insulin sensitivity and reduced inflammation (Chen et al., 2023). Moreover, traditional Chinese medicine approaches that target gut microbiota have demonstrated potential in restoring microbial balance and improving metabolic health, suggesting that a multi-targeted strategy may be beneficial in managing obesity and its comorbidities. Examples include Gegen Qinlian decoction (a four-herb formula), the single compound berberine (an alkaloid from Coptis chinensis), and acupuncture—all of which have been shown to modulate gut microbiota composition and improve metabolic parameters (Xu et al., 2025; Fan et al., 2025; Ma et al., 2025).

### 6.3 Comprehensive weight management strategies integrating genetic and microbiome information

The integration of genetic background, gut microbiota, and lifestyle factors into a comprehensive weight management strategy represents a promising frontier in personalized medicine. This multifaceted approach acknowledges that obesity is not solely a result of caloric intake and physical inactivity, but is also influenced by genetic predispositions and the complex interactions within the gut microbiome. Although specific genetic variants can influence obesity susceptibility, genome-wide association studies have revealed that known genetic loci account for less than 5% of inter-individual BMI variation. Instead, lifestyle factors and dietary choices play a more critical role in modulating gene expression through epigenetic mechanisms (Ramos-Lopez et al., 2022). Furthermore, the gut microbiota can interact with both genetic and environmental factors by affecting energy extraction from food and regulating metabolic pathways. For example, specific bacterial strains such as *Bacteroides, Prevotella*, and *Parabacteroides* have been shown to efficiently metabolize dietary polysaccharides and produce SCFAs, thereby increasing energy harvest from food and potentially exacerbating weight gain in genetically predisposed individuals (Xu et al., 2024). Pharmacological agents targeting gut microbiota (e.g., specific probiotics, postbiotics) are being investigated, but none are currently approved for obesity treatment. Multidisciplinary collaboration involving geneticists, nutritionists, microbiologists, and behavioral scientists will be essential to develop integrative models (Fernández-Pato et al., 2025).

### 6.4 Ultra-processed foods, nutritional supplements, and gut health

Long-term consumption of ultra-processed foods (UPFs) has been associated with reduced gut microbial diversity and dysbiosis (Faggiani et al., 2025). However, beyond the direction of change, it is critical to recognize that alpha diversity alone is an incomplete metric. Even when an intervention or exposure is associated with increased alpha diversity, such a finding does not automatically indicate improved gut health, because the increase may be driven by the enrichment of less beneficial or even pathogenic species. Thus, any interpretation of alpha diversity changes must examine which specific taxa are affected, rather than relying on the diversity index in isolation (Azzolino et al., 2025). This seemingly counterintuitive finding—typically increased diversity is considered beneficial—may reflect an enrichment of less beneficial or even pathogenic species rather than an improvement in gut health. Interpretation of alpha diversity changes must therefore consider which specific taxa are affected. A low-fiber diet limits the growth of beneficial short-chain fatty acid (SCFA)-producing bacteria and may contribute to gastrointestinal symptoms such as bloating and constipation (Connell et al., 2024). Furthermore, reliance on single-fiber supplements rather than a diverse array of plant fibers may not provide the same prebiotic benefits, potentially hindering proper gut function (Liu et al., 2023).

A diverse intake of carbohydrates and phytochemicals from whole plant-based foods—fruits, vegetables, whole grains, and legumes—supports beneficial bacteria such as *Bifidobacteria* and *Lactobacilli* (Coklo et al., 2020). Polyphenol-rich foods also enhance microbial diversity and functionality (González-Gómez et al., 2025). Clinical recommendations should prioritize nutrient-dense natural foods over processed products or isolated supplements. Nutritional supplements can play a supportive role but should not replace whole foods; for example, probiotic efficacy often depends on a diverse diet that provides necessary nutrients (Puga et al., 2022). In clinical practice, oral nutritional supplements (ONS) are frequently recommended for unintentional weight loss (not caused by active dieting or increased exercise, is commonly seen in individuals with chronic diseases, the elderly, or postoperative patients). However, many ONS are ultra-processed and lack fiber and phytochemicals essential for microbial diversity. Some evidence suggests that long-term reliance on ONS may lead to adverse gastrointestinal symptoms and appetite suppression (Rivero-Mendoza et al., 2023). Clinicians must balance immediate nutritional needs with potential long-term effects on gut health.

## 7 Future research directions and technological innovations

### 7.1 Individual variability and the need for large-scale data

The interplay between gut microbiota and genetic background is highly individualized, leading to variable responses to nutritional interventions. For example, probiotic supplementation (e.g., Bacillus coagulans BC99) reduces body weight with varying success (Wang et al., 2025), and distinct gut microbiota profiles are observed in patients with non-alcoholic fatty liver disease (Burz et al., 2021).

To address this variability, large-scale databases and multicenter studies are needed to identify key factors linking microbiota to metabolic health. A systematic review indicates that certain microbiota-derived metabolites are consistently altered in obesity, suggesting potential biomarkers (Ejtahed et al., 2020). Precision nutrition leveraging baseline microbiota and clinical characteristics can improve response predictability (Deehan et al., 2024). Beyond association, mechanistic studies (e.g., stable isotope tracing, gene editing, synthetic microbiomes) are required to establish causality between specific microbial taxa and metabolic outcomes (Hul and Cani, 2023).

### 7.2 Technological innovations and standardization

Advancing precision nutrition requires integration of multi-omics (genomics, transcriptomics, proteomics, metabolomics), dynamic monitoring, and novel microbiome technologies. Multi-omics provides a comprehensive view of diet–microbiota–host interactions, enabling biomarker identification for obesity and metabolic disorders (Xu and Shi, 2025). Dynamic monitoring via wearables and mobile apps allows real-time tracking and adaptive dietary adjustments (Anchidin-Norocel et al., 2025).

Emerging technologies such as CRISPR-Cas9 and synthetic microbiomes enable targeted microbiota modulation, to ensure data reliability and cross-study comparability, standardized methods for sequencing and functional assessment are imperative. Harmonization of sequencing protocols, data processing pipelines, and bioinformatics analyses will minimize variability (Sahle et al., 2024). Functional assays measuring microbial metabolites (e.g., SCFAs) are also essential for understanding host–microbe interactions.

### 7.3 Clinical translation, regulatory frameworks, and policy support

Current evidence shows inconsistent success of probiotics, prebiotics, and dietary modifications in achieving sustainable weight loss (Boscaini et al., 2022). Well-designed trials should explore underlying mechanisms and establish standardized protocols for microbiota-targeted therapies. Multicenter studies across diverse populations are necessary to refine intervention strategies based on individual microbiota, diet, and genetics. Concurrently, ethical, privacy, and regulatory frameworks must be established. Patients need assurance that genetic and microbiome data are confidential. Regulatory bodies should develop safety and efficacy guidelines for microbiota-based interventions, including long-term impact assessments. Policymakers must address socioeconomic barriers to ensure equitable access, particularly for populations disproportionately affected by obesity.

## 8 Conclusions

This narrative review has examined the associations between gut microbiota and body weight regulation, focusing on mechanisms involving energy metabolism, immune function, appetite control, and genetic/epigenetic interactions (Table 1). While compelling correlational evidence links gut microbiota composition to obesity, causal relationships in humans remain incompletely established. Precision nutrition approaches that integrate individual microbiota, genetic, and metabolic profiles hold theoretical promise, but current evidence does not support routine clinical application. AI and multi-omics technologies offer powerful tools for hypothesis generation and data integration, yet they face substantial challenges related to validation, generalizability, ethics, and standardization. Future progress will require rigorous RCTs, method standardization, and interdisciplinary collaboration. The broad application of gut microbiota-informed precision nutrition has potential to improve individualized weight management, but this potential will only be realized through careful, evidence-based translation.

**Table 1:** Summary table.

Category	Key conclusions	Evidence & limitations
Energy extraction & metabolism	Microbiota ferments fiber into SCFAs, affecting energy extraction. Obesity linked to reduced diversity and altered phylum ratios.	Based on human observational and animal studies; causality not fully established.
Immunity, inflammation & appetite	Dysbiosis increases gut permeability, leading to chronic inflammation and insulin resistance. Metabolites regulate appetite via gut-brain axis.	Strong associations; interventions show improved inflammation and satiety.
Host interactions & precision interventions	Genetic/epigenetic factors interact bidirectionally with microbiota. Personalized diets/supplements improve metabolism but responses vary.	Genetic loci explain limited BMI variation; small-scale trials; more validation needed.
Technology & clinical translation	AI and multi-omics can predict dietary responses but lack standardization/clinical validation. Ultra-processed foods and some supplements may harm microbiota.	Mostly methodological; large, long-term RCTs needed; prioritize whole foods.
